# Labor Costs, Market Environment and Green Technological Innovation: Evidence from High-Pollution Firms

**DOI:** 10.3390/ijerph17020522

**Published:** 2020-01-14

**Authors:** Rui Gong, Yong-Qiu Wu, Feng-Wen Chen, Tai-Hua Yan

**Affiliations:** School of Economics and Business Administration, Chongqing University, Chongqing 400030, China; wangjiang@cqu.edu.cn (R.G.); chenfengwen@cqu.edu.cn (F.-W.C.)

**Keywords:** labor cost, green technological innovation, firm performance, market environment

## Abstract

Emerging economies face the challenge of increasing labor costs but also provide an opportunity to promote environmental governance and green development. Based on the perspectives of impetus and capability, the effects of rising labor costs and market environment on green technological innovation are investigated in this study. The empirical studies used the data of high-pollution firms in China from 2009 to 2018. Results demonstrate that rising labor costs deteriorates high-pollution firm performance, while highly competitive industries are affected more than other industries. Meanwhile, the influence of rising labor costs on green technological innovation has a threshold effect which illustrates an “inversely U-shaped” variation trend with the increase of degree of market monopoly. The labor costs will make biggest impact on the green technological innovation in the moderately concentrated market environment. Basing from these results, this study provides the following suggestions for emerging economies’ green development: Take rising labor cost as an opportunity to advance technological progress to the green direction, establish a sound market competition environment, and develop green finance to reduce the financing constraints of green technological innovation.

## 1. Introduction

**Figure 1:**
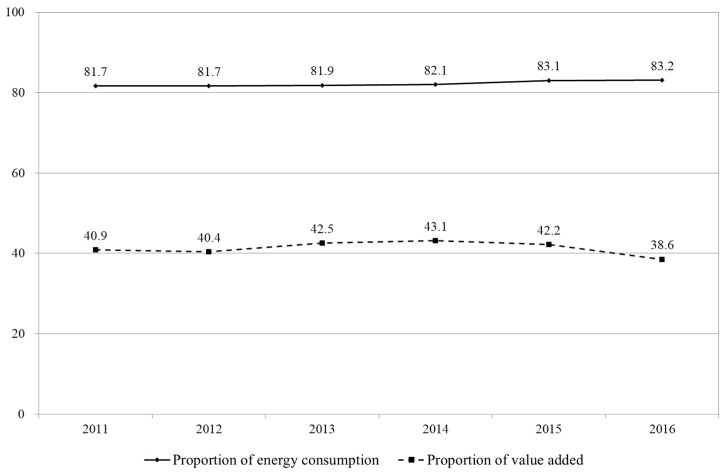
Proportion of energy consumption and added value of high pollution industry in industrial sector (%) [5].

Relying on the superiority of domestic natural resources and labor force, numerous emerging economies adopt the development pattern of high input and low technology, causing prominent energy consumption and environmental pollution. Emerging economies has become an engine of global economic growth, but it is also the primary contributor to high world energy consumption and CO_2_ emission [1]. As the largest emerging economic, China has an average GDP growth rate of up to 9.5% since the reform and opening up in 1978. However, China’s rapid economic growth is at the cost of heavy resource input and the destruction of the ecological environment [2]. China has become the world’s biggest energy consumer. Its primary energy consumption in 2017 has reached 3278.2 million tons of crude oil, accounting for 24.26% of the world [3]. China’s ecological environmental problems mainly concentrate on high-pollution industries^.^ High pollution industries in this paper refer to both high energy consumption and high pollution industries. Ministry of Ecology and Environment of China promulgated *the Listed Company Environmental Protection Check List of Industry Classification Management* (2008) [4] according to the energy consumption and environment pollution of each industry, and defined 16 industries as high polluting. These industries include thermal power, steel and iron, cement, electronic aluminum, coal, metallurgy, chemical, petrochemical, building materials, paper making, brewing, pharmaceuticals, fermentation, textiles, leather and mining industry. As shown in Figure 1, the energy consumption of 16 high-pollution industries in China accounts for more than 80% of the industrial sector, and the proportion of energy consumption in 2016 reached 83.2%, whereas the proportion of value added was only 38.6%. Therefore, reducing the energy consumption and pollution of high-pollution industries is essential to solve China’s environmental problems.

**Figure 2:**
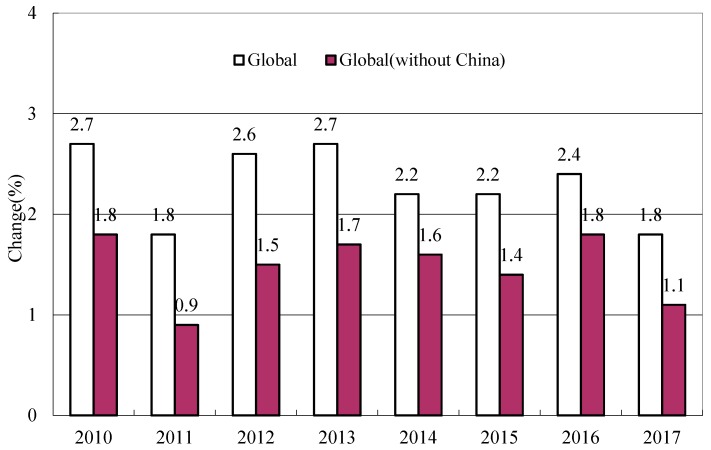
Annual average global real wage growth. [6].

Rapid economic growth in emerging economies has led to rising wages and incomes. Under the recent increasing challenges faced by the global economy owing to the global economic and trade disputes, the wages in emerging economies maintain a rapid growth rate, while wage growth decreases in many developed countries. In particular, the wage in China presents a growth against the trend. The International Labor Organization (ILO) reports that the annual growth rate of the average wage in China has reached 8% from 2008 to 2017. The actual wage growth in 136 economic entities worldwide was 1.8% in 2017, with 0.7% from China (Figure 2).

Currently, debates over the advantages and disadvantages of rapid wage growth remain. On the one hand, the rise of the production cost may bring a significant pressure on firms’ development [7]. The production costs of firms may increase if the wage growth is not attributed to an improvement in productivity [8]. Thus, firms may encounter difficulties against further development [9]. Numerous labor-intensive firms that are dominated by export processing in emerging economies have slender profits, while the rise of labor costs may weaken the competitiveness of products from the country [10]. On the other hand, emerging economies shall not rely on the benefits of labor cost to realize sustainable economic development owing to the “low wage and low productivity” trap [11]. The rise of labor costs is conducive to promoting the survival of the fittest firms, thereby forcing them to implement technological and industrial innovations. This strategy helps firms to realize the development mode of “high labor cost, high technological level, and high productivity” [12,13].

Numerous studies have reported the influences of labor costs on technological innovation [14]. The neoclassical economic theory pointed out that the increase of labor factor price may encourage firms to reconstruct their production process and technologies to reduce the investment costs of labor factors [15]. The theory of induced innovation posits that the rise of labor costs, relative to the cost of capital, may induce firms to adopt labor-saving technological innovations [16]. The human capital theory holds that, low wage prevents workers to invest in the human capital. Knowledge accumulation and technological innovation can substitute the investments in traditional production factors [17]. Therefore, the rise of labor costs facilitates the substitution of labor factors with capital and technological factors, thereby driving firms to the capital-intensive and technological-intensive paths of development [18,19]. Most empirical studies have demonstrated that the rise of labor cost promotes technological innovation in firms [20].

Nowadays, an authoritative or deterministic concept of firm green technological innovation is lacking. The earliest scholars defined it as “a general term for technologies, processes and products that reduce environmental pollution and save energy use” [21]. Then, some scholars defined green technological innovation as the use of new or improved processes, technologies, practices, systems, and products to eliminate or diminish damages to the environment, including technological innovation in energy conservation, pollution prevention, pollution recovery, green product design, and corporate environmental management [22,23]. Due to the externalities of green technological innovation, technology, market, system, etc. should be considered when analyzing the drivers of green innovation [24]. Existing research about the driving factors of green innovation can be categorized into two aspects [24,25,26]. The first aspect is based on the impetus of benefits and competitive advantage. Profit maximization is the ultimate goal of firms, and the potential economic return is considered as the primary driving force for firms to adopt an ecologically environmental-friendly market-driven position [27]. Green technological innovation is an important way for firms to gain a competitive advantage [28]. Possible benefits of green technological innovation include reduced cost of compliance with regulations [29], improved labor productivity and resource efficiency [30], and product differentiation [31]. These benefits can enhance the competitive advantage of firms. Green technological innovation can also create obstacles for competitors [23], and the “isolation mechanism” created by green technological innovation can preserve profit margins and allow firms to gain [32,33]. Benefiting from the cost reduction and revenue increase contributed by green technological innovation, firms can become more competitive [34]. The second aspect is from the perspective of environmental regulation. Institutional pressure is the main driving force for firms to adopt ecological behavior [35]. Institutional factors, such as government regulation, market requirements, and social expectations, have a significant impact on corporate green innovation behavior [36]. Strict environmental regulations and corresponding compliance costs will force firms to innovate green, thereby improving resource allocation and use efficiency [37]. Increasingly strengthened environmental standards will objectively motivate firms to take innovative environmental protection measures. Pressure from suppliers, consumers, and other stakeholders will also promote firms to implement green technological innovation [38,39]. The latest research mainly analyzes the driving factors of green technology innovation from the perspective of internal organizational environment, external social environment, and stakeholder factors [33,40,41].

Regarding the impact of labor costs on corporate environmental behavior, literature mainly examined the impact of increasing residents’ income on green development from the perspective of the whole society [42]. The Environmental Kuznets Curve points out that rising income can gradually overcome the problems of the ecological environment because technological progress will reduce the economic growth’s demand for energy and other natural resources and that high living standards can improve the public’s awareness of environmental protection [43]. However, there is no literature studied the impact of labor cost on green technology innovation from the perspective of firms. Some studies analyzed the significant impact of firm size and market structure on corporate green innovation from the perspective of industrial organization [44], because environmental protection activities require a huge investment of financial and human resources, and large firms have better opportunities and capabilities to reduce impacts on environment [45,46,47]. Given the externalities of technological innovation, the spillover effect must be minimized as much as possible. Technologies and market structures that are easy to control corporate spillovers are conducive to promoting corporate green innovation [48].

Overall, the study of green innovation is distributed in different disciplines, and a widely accepted theoretical framework is lacking. Green and innovative studies in the perspective of industrial organization primarily focus on the impact of firm size on corporate green innovation behavior, and less concern is given on the role of market competition. In the current context of an increasingly competitive international buyer’s market, the market competition has become an important determinant of corporate green technological innovation, and the actual impetus of green innovation behavior is difficult to reveal when competitive factors are neglected. At the same time, existing studies only focus on the “impetus” of firm technological innovation and ignore the impact of “capability” to implement green technological innovation. For high-pollution firms, business profits may be inhibited within a short period because of the long R&D cycle, high risk, and many uncertain factors of green technological innovation. Consequently, “financial constraints” may affect firms’ capability to implement green technological innovation. Therefore, firms that are under financial constraints have low investment on green technological innovation. Furthermore, such constraints are closely related to the challenges in transferring costs. If a firm can easily transfer rising costs, then it may reduce its financial constraints and consequently strengthen its capability for green technological innovation. By contrast, if a firm can hardly transfer costs, then its capability to upgrade is significantly restricted. Given that the degree of market monopoly is a key factor that determines whether a firm can transfer costs successfully [49], this factor is chosen as a constraint to study the effects of rising labor costs on green technological innovation.

The rest of this paper is organized as follows. Section 2 analyzes the effects of rising labor costs on green technological innovation from the perspective of impetus and capability. Section 3 identifies the data source and describes the research methods. Section 4 discusses the effects of rising labor costs on technology. In addition, the capability and comprehensive effect models of green technological innovation are constructed. Section 5 discusses the main research findings and presents the suggestions for emerging economies, and the Section 6 contains our conclusion.

## 2. Research Hypotheses

Corporate innovation is determined by the impetus and capability to innovate [44], and this definition also applies to green innovation. The impetus and ability of firms to innovate will be influenced by rising labor cost.

### 2.1. Capability for Green Technological Innovation

The increase in labor costs affects the green technological innovation capabilities of firms through financial performance. Corporate research and development activities require a large amount of stable funding [50]. Insufficient funding can lead to the termination or failure of research and development. In particular, green technological innovation has the characteristics of high capital investment, long payback cycles, and high risk of failure [25]. Thus, financing sources can only be based on internal funds [51], and debt financing is difficult to obtain. Therefore, firms implementing green technological innovation are under greater financial pressure than other competitors [52]. In addition, increasing labor costs will contribute to higher operating costs and lower financial performance [53], which can consequently impose financing constraints on green technological innovation. In consideration of the restraint effect of financing constraints on corporate R&D activities, rising labor costs will reduce the green technological innovation capabilities of highly polluting firms. Therefore, the first hypothesis is proposed as follows.

**Hypothesis 1** **(H1).** *Rising labor costs have a negative impact on the performance of high-pollution firms, consequently reducing their capability to green technological innovation through financing constraints.*

Meanwhile, the impact of labor cost on green technological innovation capability will be restricted by market structure. A firm in a highly competitive industry, which has no control and influence over the upstream supply and marketing of downstream products, cannot transfer costs and should assume profit loss due to rising labor costs [49,54]. Then, the reduction of business performance must influence the high-pollution firms’ capability for green technological innovation. By contrast, a firm that has a certain monopoly in the market and control or influence over the supply chain and product price can weaken the adverse effects of rising labor costs through cost transfer [55,56]. Furthermore, the capability of such firms to upgrade its technology is slightly influenced. Based on the analysis above, the second hypothesis is proposed as follows.

**Hypothesis 2** **(H2).** *The capability to green technological innovation of firms in competitive industries is more adversely affected than that of firms in monopolistic industries because the latter can reduce the financing constraints caused by rising labor cost through cost transfer.*

### 2.2. Impetus for Green Technological Innovation

The direct reason why rising labor costs will increase the impetus for green technological innovation is that rising wages will increase residents’ environmental awareness and encourage firms to promote resource-saving technological improvements [57]. Under the pressure of rising costs, potential economic returns are considered the primary driving force for firms to take green actions [27]. The impact of green technological innovation on the expected earnings of firms is specifically manifested in three aspects. First, green technological innovation can enable implementing firms to obtain a competitive advantage. The theory of competitive advantage proposes that green innovation allows firms to gain a first-mover advantage [24]. By increasing resource productivity, process changes, product innovation, or setting industry standards, firms occupy a favorable position in market competition. The benefits of green innovation are strategic and long term [31,58]. Second, green innovation strategies allow firms to reduce their emission levels below the policy requirements, which will reduce the cost of compliance with regulations [29], or increase additional revenue through pollutant disposal [31]. Last, green innovation helps firms obtain a good social reputation based on the environment. With the increasing awareness of consumers on environmental protection, green technological innovation is conducive to cater for consumers’ environmental protection needs and improve product competitiveness [41].

Combining the theory of industrial organization with Porter’s competitive advantage [24,43], the following provides an allocation framework that examines the impact of market competition environment on the driving force of green technological innovation. Impetus to green technological innovation is reflected by the potential economic return after innovation [27]. Assume that N firms with the same initial level of technology produce homogeneous products in a certain industry; the unit product production cost is C; the degree of monopoly in the industry decreases as the number N of firms increases; firms can reduce the production cost or enhance the competitiveness of their products through green technological innovation, and the input for green technological innovation is GTI. The product inverse demand function is P=a−b(Q), where a≥0,b≥0. Cournot competition is conducted between firms, that is, when a firm makes an output decision to maximize profits, it considers that the output of other firms is unchanged. Through static optimization, the optimal output (Q) and profit level (Π) of each firm can be calculated [43].

According to Porter’s theory of competitive advantage, if a company implements green technological innovation, it is expected to include increased product competitiveness, reduced production costs, and improved corporate image [33]. To reduce the model analysis, referring to the common methods of economic models, benefits such as competitiveness and corporate image can be quantified as a reduction in production costs [43]. Adopt $C^{*}$ to represent the unit production cost after green technological innovation. Without any consideration of the effects of other innovative decisions on other firms, the expected output and profits of firms who implemented green technological innovation are:

(1) $$Q^{*}=\frac{a-N\cdot C^{*}+\left(N-1\right)C}{b\left(N+1\right)}$$

(2) $$\Pi^{*}=\frac{{\left[a-N\cdot C^{*}+\left(N-1\right)\cdot C\right]}^{2}}{b{\left(N+1\right)}^{2}}-GTI$$

Output and profits of other firms are:

(3) $$Q=\frac{a-2C+C^{{}_{*}}}{b\left(N+1\right)}$$

(4) $$\Pi =\frac{{\left(a-2C+C^{*}\right)}^{2}}{b{\left(N+1\right)}^{2}}.$$

Let $\Psi \left(n\right)=-\frac{\partial \Pi^{*}}{\partial C^{*}}$ be the profit growth of a firm by reducing one unit of production cost through green technological innovation. Ψ(n) reflects the impetus of firms to implement green technological innovation. The effects of the number of firms on the impetus for green technological innovation can be expressed as

(5) $$\frac{\partial \Psi \left(N\right)}{\partial N}=\frac{2}{b{\left(N+1\right)}^{3}}\left[\left(N-1\right)\left(C-a\right)+2N\left({C-C}^{*}\right)\right].$$

From Equation (5), the changes in the degree of monopoly can influence the impetus for green technological innovation in two aspects. On the one hand, the first term enclosed in brackets in the Equation (5) is negative because a>C, thereby decreasing the profit due to the intensified competition caused by the increase in N. On the other hand, the market shares of each firm may decrease with the increase in N. Thus, the growth of the sales volume and monopoly profits of a firm that reduces his/her production costs relative to other firms is positively related to n through green technological innovation. This positive correlation is denoted by the second term in Equation (5). If $2\left({C-C}^{*}\right)\geq a-C$, then $\frac{\partial \Psi \left(N\right)}{\partial N}\geq 0$. The latter effect takes the dominant role at this point. The impetus for green technological innovation measured by monopoly profits is negatively related to the degree of monopoly of a firm. If a firm significantly reduces his/her production cost through green technological innovation and reduces his/her products’ market price lower than other firms’ production costs, then other firms may withdraw from the market. As the impact of rising labor cost on green technology innovation impetus cannot be directly tested, the hypothesis of combined effects of capabilities and impetus will be put forward in the following.

### 2.3. The Combined Effects

**Figure 3:**
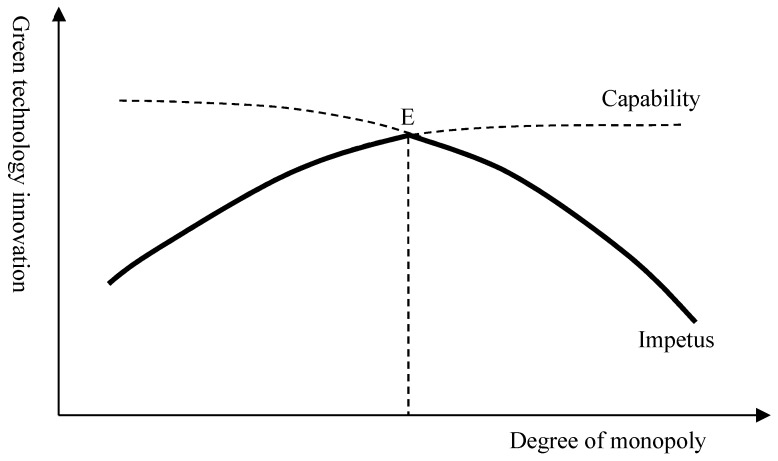
The moderating effect of market structure on green technology innovation.

The previous analysis shows that the innovation incentive under competitive conditions is stronger than that under monopolistic conditions [58,59,60,61,62]. However, the increase in labor costs can lead to a decrease in corporate profits and corporate technological innovation capabilities. Hence, the market structure’s regulatory effect on firms’ implementation of green technological innovation will no longer be linear. Figure 3 illustrates the role of the market structure in adjusting the effects of the rise of labor costs on green technological innovation. The horizontal axis is the degree of monopoly that reflects market structure, and the degree of monopoly increases as it moves to the right. The horizontal axis is the effect of the rise of labor cost on green technological innovation. The green technological innovation capability curve moves upward, whereas the impetus curve declines. These two curves exhibit an intersection point, which is Point E. On the left of Point E, the impetus for green technological innovation is stronger than capability, while capability determines or restricts whether firms will implement green technological innovation. Thus, the degree of monopoly should be increased to promote green technological innovation. On the right of Point E, the impetus of green technological innovation is weaker than capability, which may determine whether firms will innovate. Thus, increasing the degree of monopoly can decrease the impetus for green technological innovation. Accordingly, two hypotheses presented here illustrate how labor cost effects to green technological innovation.

**Hypothesis 3** **(H3).** *Increasing labor costs will increase the impetus for firms to implement green technological innovation but reduce their green technological innovation capability. The overall effect is constrained by the market environment.*

**Hypothesis 4** **(H4).** *Rising labor cost can influence green technological innovation among high-pollution firms and thus presents an “inversely U-shaped” variation trend with the increase in the degree of market monopoly. A market structure with excessive competition and monopoly restricts high-pollution firms to implement green technological innovation. By contrast, rising labor costs can promote green technological innovation because it is implemented in a market environment with balanced competition intensity.*

## 3. Data and Variable Declaration 

### 3.1. Research Data

The Ministry of Ecology and Environment of the People’s Republic of China has promulgated the “*Listed Company Environmental Protection Check List of Industry Classification Management* (2008)” [4] to define the 14 high-pollution industries, which were further divided into 16 in 2010. Considering that only mainland listed firms in the high-pollution industries will disclose data on environmental responsibility in their social responsibility reports, this paper selects 2009–2018 mainland China A-share listed firms in Shanghai and Shenzhen exchanges as the research sample. After eliminating the missing samples of R&D investment and comprehensive energy consumption data, the remaining samples are 450 samples from 45 firms. The original data are obtained from the China Wind database and social responsibility report of listed firms. The screening process of sample firms is shown in Table 1.

### 3.2. Variable Declaration 

An empirical study on relevant variables and data specification was introduced as follows.

Green technological innovation (GTI): Referring to the existing literature [39,63], the ratio of R&D investment and energy consumption is taken as the main measure of green technological innovation. The larger the ratio is, the higher the degree of green technological innovation is.

(6) $$GTI=\frac{\;R\&D\;investment}{Energy\;consumption}$$

Return on assets (ROA)**:** ROA refers to the management benefits of high-pollution firms within a certain operation period, which reflects the financial performance of high-pollution firms. ROA is used as a dependent variable in this study.

(7) $$ROA=\frac{\;Net\;profits}{Average\;balance\;of\;total\;assets}\times 100\%$$

Labor cost: According to the regulations of ILO, labor cost is the sum of wages paid by high-pollution firms to workers for the latter’s social labor services [64]. The range of labor cost is larger than that of wage. Furthermore, labor cost covers wages and salaries in currency, as well as material or nonmaterial welfares including social insurance. In this study, the labor cost was calculated by workers’ wage data in the financial statements of a high-pollution firm.

(8) $$Labor\;Cost\;=\frac{\;Total\;Payroll}{Employee\;population}$$

Market structure: The intensity of competition within an industry is measured with the Herfindahl–Hirschman Index (HHI), which is a threshold variable in the empirical study. HHI is measured by the percentages of market competition subjects in an industry over the total shares of the industry. As a comprehensive index that measures industrial concentration, its value ranges between 0 and 1. A high HHI value indicates high market concentration. HHI = 1 at a complete market monopoly. A small HHI value reflects that the market concentration is low and the market competition is intensive.

(9) $$HHI={\sum_{i=1}^{N}\left(\frac{Y_{i}}{T}\right)}^{2}$$

where $T=\sum_{i=1}^{N}Y_{i}$. N is the total number of high-pollution firms in an industry, and Y is the gross revenues of high-pollution firms.

Except for the variables listed above, the following control variables will also be used in the experimental study:

Asset scale: Due to the scale merits, the size of a company’s assets will affect its financial performance [65]. Some studies also found that, the larger the size is, the stronger the impetus for the company to implement green technological innovation [45,66]. This variable is measured by the total assets and uses the natural logarithm in the regression analysis.

Asset–liability ratio: The debt ratio of a firm will affect the operation. According to financial theory, the higher the debt ratio, the greater the fluctuation of corporate performance [67]. From a capability perspective, firms with high debt ratios have a weak capability to implement green technological innovation. From a risk perspective, firms with high debt ratios have a low chance of risking technological innovation [68]. This variable is calculated as follows:

(10) $$Asset-liability\;ratio=\frac{\;Total\;liability}{Total\;assets}\times 100\%.$$

Liquidity of assets: according to financial theory, the stronger the liquidity of a firm’s assets, the better it is for improving financial performance [69]. However, from the perspective of technological innovation, given that technology is reflected in fixed and intangible assets, the greater the proportion of firms’ current assets, the lower the foundation and potential of technological innovation. The liquidity of assets calculated as follows:

(11) $$Liability\;of\;assets=\frac{\;Current\;assets}{Total\;assets}\times 100\%.$$

Top 10 shareholders: the higher the proportion of the top 10 shareholders’ equity, the more concentrated the equity, but whether the concentration of equity improves financial performance remains controversial [70,71]. Similarly, equity concentration may also affect green technological innovation [72]. This variable is calculated as follows:

(12) $$Top\;10shareholders=\frac{\;Shares\;held\;by\;the\;top\;10\;Shareholders}{Total\;shares}\times 100\%.$$

Capital cost: High financing costs will reduce corporate financial performance [73]. Financing cost will influence corporate green technological innovation as well, because higher financing cost is detrimental to firms’ green technological innovation [52]. This variable is calculated as follows:

(13) $$Capital\;cost=\frac{\;Interest\;cost}{Interest-bearing\;liabilities}\times 100\%$$

Tax burden: Heavy tax burden will adversely affect corporate financial performance [74]. Tax burden is used as a control variable for financial performance in this study.

(14) $$Tax\;burden=\frac{\;Total\;tax\;payment-Government\;subsidy}{\;Operating\;revenues}\times 100\%$$

Table 2 lists the description statistics of all variables.

## 4. Estimation of Parameters

### 4.1. Estimation of Threshold Effect of Labor Cost on Firms Performance

To test Hypothesis 1 in Section 2, regression model on return on assets (ROA) is established based on variables selection in Section 3 as follows:

(15) $${ROA}_{it}=\alpha_{0}+\alpha_{1}labor{\;cost}_{it}+\sum_{k=1}\rho_{k}X_{it}+\varepsilon_{it}$$

where Labor cost is the independent variable, X are control variables for ROA described in Section 3.2, including asset scale, asset-liability ratio, liquidity of assets, top 10 shareholders, capital cost, tax burden, and HHI. $\rho_{k}$ is the estimated coefficient of the kth control variable, and $\varepsilon_{it}$ is the residual error.

First, the OLS method was used to estimate the parameters (Model 1 in Table 3), and the panel data were tested for varying intercept models. The F statistic was 8.03, and the assumption of constant intercept was rejected. The variable intercept model of panel data includes fixed-effect and random-effect models. The Hausman test results indicate that fixed-effect models should be used. From the parameter estimation results of Model 2 in Table 3, the coefficient of labor cost is generally negative, which passes through the 5% significance test, which shows that rising labor costs cause the financial performance to deteriorate among the high-pollution firms in China. Asset scale, liquidity of assets, and HHI are positively related to ROA. Asset-liability ratio and capital cost are negatively related to ROA. The relationship between Top 10 shareholders and Tax burden and ROA is not significant.

In Hypotheses 2 and 4, the market structure is a variable that adjusts the relationship between labor cost and green technological innovation. Several empirical studies have applied paying by term to estimate the adjustment effect [66]. The adjustment effect of market structure presents an “inversely U-shaped” variation. However, paying by term is a method based on the hypothesis of linear relationship, which is inapplicable in this study. Therefore, the threshold regression analysis method was applied to test Hypotheses 2 and 4 [75]. This method can determine whether a nonlinear adjusting effect is evident by verifying several threshold values.

According to Hypothesis 2, the effects of labor cost on high-pollution firm performance were verified by using a market structure as the threshold variable. Based on Equation (15), the threshold regression panel model is developed:

(16) $$\begin{array}{l} {ROA}_{it}=\alpha_{0}+\alpha_{1}labor{\;cost}_{it}I(HHI_{it}\leq \gamma_{1})+\alpha_{2}labor{\;cost}_{it}I(\gamma_{1}\leq HHI_{it}\leq \gamma_{2})\\ \;\;\;\;\;\;\;\cdot \cdot \cdot +\alpha_{n}labor{\;cost}_{it}I(\gamma_{n-1}\leq HHI_{it}\leq \gamma_{n})+\alpha_{n+1}labor{\;cost}_{it}I(HHI_{it}>\gamma_{n})\\ \;\;\;\;\;\;\;+\sum_{k=1}\lambda_{k}X_{it}+\varepsilon_{it} \end{array}$$

where I(·) is an index function, and HHI is a control and threshold variable. $\gamma_{n}$ is the threshold value. The threshold number and threshold values were tested by Bootstrap method (Table 4). The double threshold model demonstrates that the values have failed in the significance test (*p* = 0.1293), and the model has only one effective threshold, which is HHI = 0.1526.

In Table 5, Model 1 provides the threshold regression results of the effects of rising labor cost on ROA. When HHI < 0.1526, the regression coefficient ($\alpha_{1}$) is estimated as 0.0421, which passes through the 1% significance test. Thus, rising labor cost can reduce financial performance when a strong intensity of industrial competition is evident. The coefficient fails in the significance test when HHI ≥ 0.1526. Therefore, rising labor costs does not significantly influence financial performance in the market with low industrial competition level. The results of other control variables are the same as those of Model 2 in Table 3. To test the robustness of the parameter estimation results, control variables significantly correlated with labor cost (10% significance test) are removed in Model 2 of Table 5. From the new threshold regression results of Model 2 in Table 5, the threshold values and labor cost estimation coefficients slightly change, indicating that the parameter estimation results are robust. However, the coefficient of the control variable asset scale has changed considerably probably because the asset scale is significantly correlated with other control variables that have been eliminated.

### 4.2. Effects of Labor Costs on Green Technological Innovation under Different Market Structures

To verify Hypothesis 3, the following regression equation model for green technological innovation (GTI) was established based on the selection of control variables in Section 3:

(17) $${GTI}_{it}=\beta_{0}+\beta_{1}labor{\;cost}_{it}+\sum_{k=1}\eta_{k}Z_{it}+\xi_{it}$$

In the above equation, labor cost is the independent variable, Z are control variables for GTI described in Section 3.2, including asset scale, asset-liability ratio, liquidity of assets, top 10 shareholders, and capital cost. $\eta_{k}$ is the estimated coefficient of the kth control variable, and $\xi_{it}$ is residual error.

First, the OLS method was used to estimate the parameters (Model 1 in Table 6), and the panel data were tested for varying intercept models. The F statistic was 6.14, and the assumption of constant intercept was rejected. The variable intercept model of panel data includes fixed-effect and random-effect models, and the Hausman test results indicate that fixed-effect models should be adopted. Based on the parameter estimation results of Model 2 in Table 3, the coefficient of labor cost is generally positive, which passes through the 1% significance test. The result indicates that the overall labor costs can promote green technological innovation, demonstrating that rising labor costs generally promote green technological innovation for high-pollution firms. In addition, asset scale and GTI are positively correlated, indicating that the expansion of the company’s scale is conducive to promoting green technological innovation. Liquidity of assets and ROA are negatively correlated. Asset-liability ratio, Top 10 shareholders, capital cost, and tax burden are not significantly related to ROA.

According to Hypothesis 4, the effects of labor cost on high-pollution firm performance were verified by using a market structure as the threshold variable. Based on Equation (17), the threshold regression panel model can be expressed as:

(18) $$\begin{array}{l} {GTI}_{it}=\beta_{0}+\beta_{1}labort{\;cotst}_{it}I(HHI_{it}\leq \gamma_{1})+\beta_{2}labort\;{cotst}_{it}I(\gamma_{1}\leq HHI_{it}\leq \gamma_{2})\\ \;\;\;\;\;\;\;\cdot \cdot \cdot +\beta_{n}labort{\;cotst}_{it}I(\gamma_{n-1}\leq HHI_{it}\leq \gamma_{n})+\beta_{n+1}labort{\;cotst}_{it}I(HHI_{it}>\gamma_{n})\\ \;\;\;\;\;\;\;+\sum_{k=1}\eta_{k}X_{it}+\xi_{it} \end{array}$$

where I(·) is an index function, and HHI is a control and threshold variable. $\gamma_{n}$ is the threshold value.

Threshold value was estimated and tested using the Bootstrap method (Table 7). The F statistics indicates that the existence of three threshold values is impossible, but two effective threshold values exist, namely, HHI = 0.173 and HHI = 0.2145.

Model 1 in Table 8 illustrates the threshold regression results on the effects of the rise of labor costs on green technological innovation. When HHI < 0.1173, the coefficient of labor costs fails the significance test. Therefore, the rise of labor costs may not promote green technological innovation when an intensive industrial competition occurs. When 0.1173 ≤ HHI < 0.2145, the regression coefficient is 2.839498 reaches the 1% significance test. Therefore, increasing the degree of monopoly induces labor costs to exert positive influence on green technological innovation. When HHI ≥ 0.2145, the regression coefficient is 1.523217, which reaches the 1% significance test. This finding reveals that although green technological innovation has been promoted when HHI in the middle level, further increasing the degree of industrial monopoly may weaken its effect.

The results of other control variables are the same as those of Model 2 in Table 8. To test the robustness of the parameter estimation results, control variables significantly correlated with labor cost are removed in the new threshold regression model. From the new threshold regression results of Model 2 in Table 8, the thresholds of the two models remain constant, and the estimated coefficients of labor cost and other control variables slightly change, indicating that the estimation results are robust. The coefficient of *capital* *cost* has changed probably because the strong correlation between *capital cost* and other control variables.

## 5. Discussion

Under the pressure of increasing labor cost, firms’ primary goal of technological innovation is to obtain economic benefits. Thus, this paper studies the relationship among wage increases, market structure, and green technological innovation from the perspective of economic benefits. Existing literature mainly focuses on the driving force for green technological innovation but ignores the constraints of firms’ capability to implement technological innovation. We elaborated on the impact of rising labor costs on the green technological innovation of high-pollution firms from the perspective of innovation capabilities and impetus and on the moderating effect of market structure in the relationship between the two. The results of this paper are different from the existing literature:

First, rising labor costs restrict the high polluting firms’ capability to green technological innovation, and the restriction is more apparent in fiercely competitive markets than in monopolistic markets. As shown in Table 3, rising labor costs will lead to a decline in corporate profits in the short term. Although the efficiency wage theories suggest that high wages have a positive effect that higher efficiency wages than market clearing levels can significantly improve corporate performance [76,77]. The rapid increasing wages in China are not voluntarily paid by firms based on labor productivity but rather driven by compulsory government policies. The introduction of the Labor Contract Law (2008) and the Social Insurance Law (2011) by the Chinese government has increased the labor cost of firms over the growth of labor productivity. Such wage growth forced by policies is determined by the exogenous factors and is universal. Thus, it would not produce extra excitation effects on firm employees and positive effects on firm finance. The rise in labor costs in China is mainly reflected in increased operating costs, reduced financial performance of firms, and further imposed financial constraints on the implementation of green technological innovation. The empirical research results in Table 5 show that different market structures give manufacturers different cost pass-through capabilities. Firms with a higher degree of monopoly will obviously have greater cost pass-through capabilities. Hence, they will be less affected by rising labor costs. Instead, the performance of highly competitive industries will be negatively affected because costs are difficult to conduct throughout the industrial chain.

Second, rising labor costs have promoted green technological innovation in high-pollution firms. Rising labor costs have a negative effect on the financial performance of high-pollution firms and thus bring financial constraints to corporate green technological innovation. However, rising labor costs will also give high-pollution firms incentives to implement green technological innovation. The main reason is that under the background of rising labor costs, the primary goal of firm technological innovation is to reduce costs and increase economic benefits. With the characteristics of high resource input and high energy consumption in high-pollution industries, green technological innovation is an important method for high-pollution firms to reduce production costs and improve competitive advantages. Therefore, the implementation of green technological innovation by high-pollution firms can receive greater expected benefits [30]. On the basis of the results of in Table 5, China’s high-polluting industries have a stronger dynamic effect than a pressure effect by increasing labor costs, and the empirical research results show a significant positive effect.

Third, the impact of rising labor costs on greening technological innovation has an inverted “U” relationship with increasing market monopoly. From the results of in Table 8**,** under sufficient market competition, the impact of rising labor costs on corporate innovation is not significant, and the impact of green technological innovation is significantly positive only in a high degree of monopoly. In addition to the theory of industrial organization, the conclusion that monopolistic firms are capable of technological innovation proves the theoretical hypothesis in Section 2. This result is different from the view that market concentration is conducive to promoting technological innovation in firms [44,63]. This paper believes that, from the perspective of innovation momentum only, the main driving force for green technological innovation comes from the available competitive advantages of firms [31] and barriers for other competitors [23,28], but in a monopolistic market environment, the value of this competitive advantage is limited and cannot bring direct economic benefits to the company. At the same time, monopoly firms’ cost is easier to pass on facing the increasing labor cost. Thus, competitive markets are more capable of inducing firms to implement green technological innovation than monopolistic markets [59,60,61,62]. Synthesizing the different moderating effects of market structure on labor cost on green technological innovation capability and impetus, this paper proposes and validates this new view: The impact of labor cost on green technological innovation with rising market monopoly should be represented as a “U” relationship.

Emerging economies will inevitably face the pressure of rising labor costs during rapid economic growth. At this time, the development concept of “polluting first and then treating” must be changed to take advantage of rising labor costs as an opportunity to promote technological innovation and industrial transformation. Otherwise, the “extensive” development model with high investment, high pollution, and low technology will inevitably fail to achieve sustainable economic development. Rising labor costs may either be a driving factor for green technological innovation or a constraint that hinders green technological innovation. Policy implications for emerging economies from the findings of this paper include the following:

The first is to seize the opportunity to implement green technological innovation with rising labor costs. With rising wages and higher consumption levels, people’s awareness of the ecological environment will gradually increase. The implementation of green technological innovation by firms will not only help meet the environmental protection needs of consumers but also reduce costs and gain competitive advantages through innovation. Therefore, this stage of development is critical for emerging economies to change the development approaches of “grow first, clean up later”. The government needs to guide the direction of firm technological development. The firm’s technological progress can be developed in a resource-saving and environmental-friendly direction through government’s encouraging, support and system regulation, and green sustainable development can be achieved through industrial transformation and production environmental problems solving. After World War II, taking the industrial structure of advanced countries in Europe and the United States as examples, steel, shipbuilding, heavy chemical industry, automobiles, electrical machinery, etc. were selected as leading industries. Although it created Japanese economic miracles, “heavy industry and light environmental protection” caused major environmental pollution problems. In the 1970s, Japan began to transform its development model. By supporting comprehensive measures, such as green technological innovation, adjusting investment structure, and strengthening environmental legislation, it successfully realized the industry’s green development and entered the ranks of high-income countries.

The second is to create a good market competition environment. Neither excessive competition nor excessive monopoly is the optimal market structure to promote green technological innovation. Japan’s Ministry of International Trade and Industry proposed the “new industrial system theory” in 1963, arguing that Japanese firms at that time were generally small in terms of production and operation scales. Many small firms were involved in product prices, technological improvements, and equipment investment. However, they were stuck into excessive competition and incapable of competing with foreign firms. Therefore, the government should encourage firms to expand their scale, avoid excessive competition, and consciously promote cooperation and shareholding among firms. The Japanese government issued a policy to support the appropriate concentration of the market to improve the competitiveness and innovation of firms. For industries that are over-competitive, the government’s support strategy should accelerate the emergence of monopolistic firms and advantageous firms. Only under the concept of green development can the comparative advantages of global industrial division of labor be seized to occupy the high ground of the value chain. Of course, excessive monopoly is also not an ideal market structure because it may reduce the enthusiasm of high-polluting firms for green technological innovation.

The third is to reduce the constraints of green technology financing of firms. Rising labor costs have brought financing constraints to firms’ green technological innovation capabilities. Given its high investment and high risk, green technological innovation is inseparable from good financial environment support [52]. The experience of developed countries shows that the development of green finance requires the cooperation between the government and financial institutions and that diversified green financial products allow financial support and risk prevention for corporate green technological innovation. The government can increase the enthusiasm of financial institutions for green technological innovation through fiscal discounts and tax incentives, and strengthen the supervision of using funds in highly polluting industries to avoid the extensive development of high-pollution firms’ funds for capacity expansion.

## 6. Conclusions

Many emerging economics have achieved rapid economic development by taking advantage of their low labor costs and “grow first, clean up later” approaches. However, such development mode is not sustainable. The labor cost must be increased after reaching a certain stage of economic development, which may “force” high pollution firms to implement green technology innovation. The effects of the rise of labor costs on green technological innovation from the perspectives of the firms’ capability and impetus are analyzed in this study. On the one hand, the rise of labor costs can affect the green technological innovation capability of high-pollution firms. The constraint on the green technological innovation capability is strengthened by intensifying market competition. On the other hand, the rise of labor costs can increase the green technological innovation impetus of high-pollution firms. The competitive market can induce high-pollution firms to implement green technological innovation better than their monopoly market counterparts.

An empirical study based on the data of listed high-pollution firms in China from 2009 to 2018 is conducted. Findings reveal that the rise of labor costs can exert negative effects on the financial performance of high-pollution firms. Moreover, the rise of labor cost influences the competitive industries more compared with the monopoly industries. The influence of rising labor costs on green technological innovation has a threshold effect. The effects of the rise of labor costs on the green technological innovation of high-pollution firms illustrate an “inversely U-shaped” variation trend with the increase of degree of market monopoly. An insignificant relationship exists between labor cost and green technological innovation among industries in a highly competitive market. The labor costs will make biggest impact on the green technological innovation in the moderately concentrated market environment. Moderately concentrated is the best market competition environment. The influence of rising labor costs on green technological innovation will weaken in over-competitive and over-concentration market environment.

This paper provides development suggestions for emerging economies, including taking the rising labor costs as an opportunity to advance technological progress to the green direction, establishing a moderately concentrated market competition environment, and reducing the financial constraints on green technological innovation through the development of green finance.

## Figures and Tables

**Table 1:** Screening process of sample firms.

Sample Processing Method	Number of Firms	Sample Size
A-share listed firms in high pollution industries	1023	8123
Exclude firms with incomplete or missing R&D investment data	721	4967
Exclude firms with incomplete or missing comprehensive energy consumption data	45	450
Valid samples of remaining firms	45	450

**Table 2:** Description of variables and statistics. GTI: green technological innovation; ROA: return on assets; HHI: Herfindahl–Hirschman Index.

Variables	Unit	Mean	Standard Deviation	Minimum	Maximum
GTI	10,000 CNY/kwh	0.0584	0.1768	0.0015	8.9525
ROA	%	2.9334	5.6761	−32.632	28.169
Labor cost	1000 CNY	98.868	44.293	25.270	342.151
HHI	--	0.1002	0.1039	0.0190	0.5302
Asset scale	CNY	1.08 × 10^10^	2.25 × 10^10^	6.73 × 10^8^	1.90 × 10^11^
Asset-liability ratio	%	48.189	17.104	7.1145	111.23
Liquidity of assets	%	44.797	14.906	13.350	92.068
Top 10 shareholders	%	54.896	15.025	19.932	91.697
Capital cost	%	8.9140	7.0208	1.0763	29.778
Tax burden	%	2.5184	2.8915	−10.789	22.509

**Table 3:** Parameter estimation of labor cost impact on financial performance.

Independent Variables	Dependent Variable: ROA	
	Model 1: OLS	Model 2: FE
Labor cost	−0.0012 * (−1.76)	−0.0023 ** (−2.09)
Asset scale	1.8097 *** (25.13)	1.264863 *** (4.77)
Liquidity of assets	0.0415 *** (8.77)	0.1015474 *** (9.00)
Asset–liability ratio	−0.2235 *** (−239.29)	−0.2279553 *** (−246.26)
Top 10 shareholders	0.1442 ** (2.48)	0.0258974 (2.81)
Capital cost	−0.1865 *** (−13.04)	−0.104984 *** (−4.51)
Tax burden	0.0001 (0.38)	−0.0003984 (−1.45)
HHI	−3.9542 *** (−3.71)	15.49862 ** (2.44)
Constant	−31.5747 *** (−19.27)	−18.99597 *** (−3.09)
R-squared	0.9231	0.9070
F-statistics	7493.28	8183.14
Hausman test		48.04
Number of observations	450	450

t Statistic is listed in parentheses; *, **, and *** denote the levels of significance test by 10%, 5%, and 1%, respectively; Stata software was used for estimation in this study, hereinafter.

**Table 4:** Estimation and test of threshold value of firm performance equation.

Threshold Number	Threshold Value	F-Statistic	*p*-Value
Single	(0.1526)	121.720 ***	0.0000
Double	(0.1526, 0.2791)	20.708	0.1293

*** Denote the levels of significance test by 1%.

**Table 5:** Threshold regression of labor cost impact on financial performance.

Independent Variables	Dependent Variable: ROA	
	Model 1: OLS	Model 2: FE
$\alpha_{1}$ (HHI < $\gamma_{1}$)	−0.0421 *** (−9.51)	0.0434 *** (2.80)
$\alpha_{2}$ (HHI ≥ $\gamma_{1}$)	0.0001 (0.04)	−0.0004 (−0.58)
Asset scale	1.000 *** (3.85)	11.430 *** (10.05)
Liquidity of assets	0.0871 *** (7.88)	−0.229747 *** (−255.64)
Asset–liability ratio	−0.2281 *** (−253.63)	
Top 10 shareholders	0.0227 *** (2.54)	
Capital cost	−0.0868 *** (−3.83)	−0.0857455 *** (−3.75)
Tax burden	−0.0004 (−1.52)	−0.0004374 (−1.59)
HHI	15.5673 ** (2.52)	
Constant	−15.5279 ** (−2.44)	13.92737 *** (60.84)
$\gamma_{1}$	0.1526	0.1526
R-squared	0.9099	0.8222
F-statistics	6930.55	3165.93
Number of observations	450	450

t Statistic is listed in parentheses; ** and *** denote the levels of significance test by 5% and 1%, respectively.

**Table 6:** Parameter estimation of labor cost impact on green technological innovation.

Variables	Dependent Variable: GTI	
	Model 1: OLS	Model 2: FE
Labor cost	0.000182 *** (76.35)	0.000155 *** (58.14)
ln(asset scale)	0.018094 *** (13.26)	0.007116 ** (1.92)
Liquidity of assets	−0.002146 *** (−23.80)	−0 0.001266 *** (−6.56)
Asset–liability ratio	1.90 × 10^−6^ (0.11)	6.48 × 10^−6^ (0.41)
Top 10 shareholders	0.000096 (0.88)	0.000119 (0.76)
Capital cost	0.000287 (1.05)	−0.000149 (−0.37)
Constant	−0.259107 *** (−8.30)	−0.0565547 (0.56)
R-squared	0.5753	0.5045
F-statistics	127.74	492.47
Hausman test		110.92
Number of observations	450	450

t Statistic is listed in parentheses; ** and *** denote the levels of significance test by 5% and 1%, respectively.

**Table 7:** Estimation and test of threshold value of technological upgrading equation.

Threshold Number	Threshold Value	F-Statistic	*p*-Value
Single	(0.1529)	1071.75 ***	0.000
Double	(0.1173, 0.2145)	4715.02 ***	0.000
Triple	(0.0793, 0.1173, 0.2145)	16.16	0.6167

*** Denote the levels of significance test by 1%.

**Table 8:** Threshold regression of labor cost impact on green technological innovation.

Variables	Dependent Variable: GTI	
	Model 1: OLS	Model 2: FE
$\beta_{1}$ (HHI < $\gamma_{1}$)	9.12 × 10^−7^ (0.46)	2.80 × 10^−7^ (0.14)
$\beta_{2}$ ($\gamma_{1}$≤ HHI < $\gamma_{2}$)	0.000284 *** (191.00)	0.000284 *** (184.99)
$\beta_{3}$ (HHI ≥ $\gamma_{2}$)	0.000152 *** (98.47)	0.000152 *** (95.39)
ln(asset scale)	0.016276 *** (8.82)	
Liquidity of assets	−0.000838 *** (−10.57)	
Asset–liability ratio	−2.12 × 10^−6^ (−0.33)	−9.91 × 10^−6^ (−0.02)
Top 10 shareholders	−0.000028 (−0.43)	
Capital cost	−0.000056 (−0.34)	−0.000420 ** (−2.53)
Constant	−0.262619 *** (−6.79)	0.055301 *** (33.19)
$\gamma_{1}$	0.1173	0.1173
$\gamma_{2}$	0.2145	0.2145
R-squared	0.7001	0.6420
F-statistics	185.43	168.47
Number of observations	450	450

t Statistic is listed in parentheses; ** and *** denote the levels of significance test by 5% and 1%, respectively.
